# Diaphyseal and Metaphyseal Modeling Defects—Clinical Findings and Identification of WRAP53 Deficiency in Craniometadiaphyseal Dysplasia

**DOI:** 10.3389/fgene.2021.684905

**Published:** 2021-08-12

**Authors:** Yun Hao, Xiao-Lin Wang, Jun Xiao, Chun-Lei Jiao, Xin-Yao Meng, Jia-Chao Guo, Jing-Fan Shao, Jie-Xiong Feng, Jin-Peng He

**Affiliations:** ^1^Department of Radiology, Tongji Hospital, Tongji Medical College, Huazhong University of Science and Technology, Wuhan, China; ^2^Department of Pediatric Surgery, Pediatric Orthopedic, Tongji Hospital, Tongji Medical College, Huazhong University of Science and Technology, Wuhan, China

**Keywords:** diaphyseal and metaphyseal modeling defects, craniometadiaphyseal dysplasia, WRAP53 deficiency, osteoporosis, limb deformity

## Abstract

**Background:** Diaphyseal and metaphyseal modeling defects lead to severe changes in bone mass and shape, which are common features in osteoporosis that linked to non-vertebral fractures. Original mechanism of diaphyseal and metaphyseal modeling defects has proved elusive. Studying rare syndromes can elucidate mechanisms of common disorders and identify potential therapeutic targets.

**Methods:** We evaluated a family pedigree with craniometadiaphyseal dysplasia (CRMDD, OMIM 269300), a genetic disorder that is characterized by cortical-bone thinning, limb deformity, and absent of normal metaphyseal flaring and diaphyseal constriction. Systemic radiographic examination and serum hormone test were made for this rare disease. One patient and her two normal parents were examined by means of whole-exome sequencing (WES) to identify the candidate pathogenic gene and rule out mucopolysaccharidosis and Prader–Willi Syndrome by means of Sanger sequencing.

**Results:** There are several conspicuous radiographic characteristics: (1) bullet-shaped phalanges, (2) long and narrow pelvic inlet, absent of supra-acetabular constriction, (3) round rod-shaped long tubular bones, (4) prominent aiploic mastoid, (5) bending-shaped limb, genua varus and genu varum, and (6) congenital dislocation of elbow. Here, we did not find any wormian bones, and there are several typical clinical characteristics: (1) macrocephaly and wide jaw, (2) Avatar elf-shaped ears, pointed and protruding ears, (3) hypertrophy of limbs, (4) flat feet and giant hand phenomenon, (5) nail dystrophy, (6) limb deformity, (7) high-arched palate, (8) superficial hemangiomas, (9) tall stature, and intellectual disability. In this patient, we found biallelic frameshift deletion mutations in WRAP53, and those two mutations were transmitted from her parents respectively.

**Conclusions:** We describe her clinical and radiological findings and presented a new subtype without wormian bones and with a tall stature. Our study showed that craniometadiaphyseal dysplasia was caused by a deficiency of WRAP53 with autosomal recessive inheritance.

## Introduction

A rare disease is defined as one that affects fewer than 200,000 people in the US or <1 in 2,000 people in Europe (Boycott et al., 2017). Over 6,000 rare disorders affect ~1 in 10 Americans. Rare genetic bone disorders remain the major causes of disability (Faruqi et al., 2014). Studying the severe phenotypes of rare syndromes can help to elucidate the pathogenesis of common diseases and identify potential therapeutic targets. Lessons from rare bone diseases contributed to the development of the most successful class of bone active agents, the bisphosphonates (Bassett et al., 1969; Fleisch, 1997, 2002). Recent researches on rare bone diseases have elucidated the key pathways of bone resorption and bone formation, and identified new targets, including cathepsin K (Costa et al., 2011; Drake et al., 2017; Lotinun et al., 2019) and sclerostin (Li et al., 2009; Papapoulos, 2011; Ominsky et al., 2017), for which drugs are now in clinical trials (Cheng et al., 2020). Although many of these rare bone diseases have very low incidence rate, the total number of affected persons is quite large. At present, there is no effective treatment; however, significant promising advances in therapeutic modalities were developed that will limit patient suffering and treat their skeletal disabilities.

Cortical bone fragility is a common feature of osteoporosis, which is related to non-vertebral fractures (Hughes et al., 2019). A whole-genome sequencing (WES) study showed that Pyle's disease was caused by sFRP4 deficiency, that cortical bone and trabecular bone homeostasis were regulated by different mechanisms, and that sFRP4-mediated cross-regulation of Wnt and BMP signaling was essential to obtain proper cortical bone thickness and stability (Kiper et al., 2016; Chen et al., 2019). A study of this rare Pyle's disease found a new regulatory mechanism of bone metabolism, which made an important innovation for the treatment of osteoporosis.

Craniometadiaphyseal dysplasia (CRMDD, OMIM 269300) is characterized clinically by macrocephaly with frontal prominence, dental hypoplasia, and increased bone fragility (Dhar et al., 2010). Diagnostic radiologic features include thin bone in the upper part of the calvaria with prominent wormian bones, diaphyseal widening of the long tubular bones in early childhood with wide undermineralized metaphyses in older individuals, widened ribs and clavicles, and broadening of short tubular bones with increased transparency and thin cortices (Langer et al., 1991; Dhar et al., 2010). Craniotubular bone dysplasia is a hereditary disease characterized by abnormal modeling of the skeleton and moderate sclerosis of the calvarium and base of the skull (Dhar et al., 2010). However, molecular diagnosis using existing technology and knowledge remains a challenge, and this rare disease is still a therapeutic challenge for clinicians because of the lack of understanding of the underlying mechanisms. After searching Pubmed, Web of Science database, and OMIM website, only four literatures and four cases of this disease were reported as CRMDD in total (Schwarz, 1960; Langer et al., 1991; Santolaya et al., 1998; Dhar et al., 2010). Parental consanguinity in the families with CRMDD reported by Langer et al. (1991) and Santolaya et al. (1998) is consistent with autosomal recessive inheritance. None of them revealed a candidate gene for CRMDD. Here, we reported the WES result for the first time. We also first conducted a comprehensive radiographic study of this rare disease, and we revealed this second type of CRMDD that is without prominent wormian bones.

## Methods

### Patient Presentation and Clinical Characteristics

#### Patient

**Figure 1:**
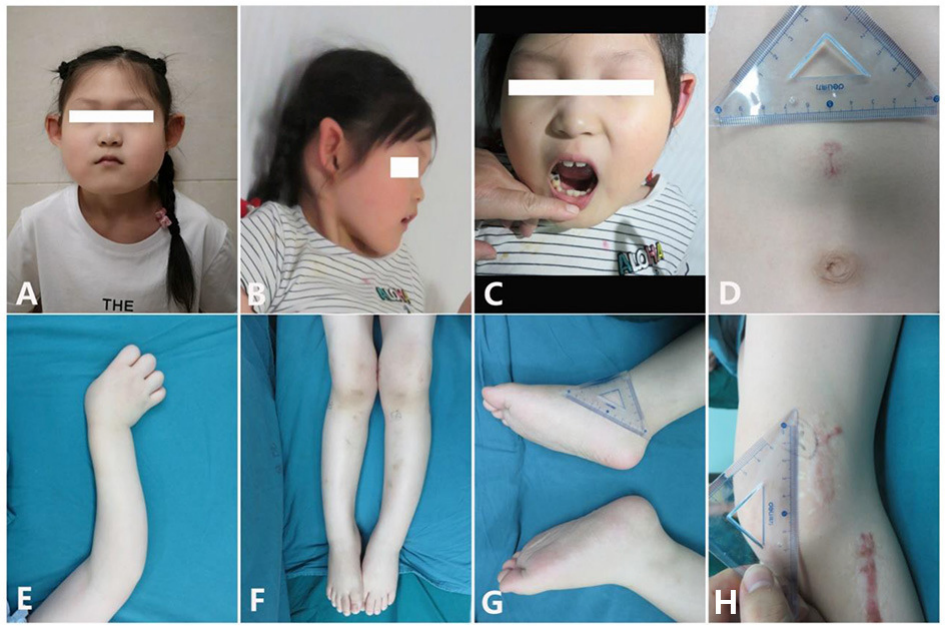
Clinical characteristics of the patient [age 6 years and 6 months; **(A,B)** Facial features: small mouth with thin lips, pointed ears with no obvious earlobe; **(C)** sparse and irregular teeth; **(D)** superficial hemangioma; **(E)** tortuous arm with ulnar deviation of her wrists; **(F)** genu valgum; **(G)** big feet and flat feet; **(H)** scar hyperplasia].

The patient was referred for skeletal deformity at 2 years and 7 months. She received a bilateral epiphyseal blocking operation because of her severe O-type legs, and the implants were removed 1 year later. However, she received epiphyseal blocking operation at 4 years and 11 months again and with one of the implants removed in the last year. Now she came here to remove the other implants. Her legs were still abnormal in appearance and her arms were also tortuous in some extents. She has a problem of complete elbow extension and an ulnar deviation of her wrist joints. Her fingers look like swelling and with the end dactylus tapered off to a point. She has big feet and hands than other children at the same age. At the age of 6 years, her height was 141 cm (>97th), and her weight was 39.6 kg (>97th). However, she developed without precocious puberty. Her total foot length was 19.5 cm. She has brachydactyly and flatfoot. There is a superficial hemangioma located above her umbilicus since birth. Her facial features included a small mouth with thin lips, flat forehead, macrognathia, and a pointed chin. Her teeth developed small and sparse. Her two pointed ears have no obvious earlobe (Figure 1). The patient was born via uterine-incision delivery with a huge body weight at 5.4 kg (>97th) and with a gestational age at 37 weeks and an Apgar score of 8. Her ears had different shapes as others at her birth. During infancy and childhood, she exhibited normal psychomotor development but had an accelerated linear growth in her height. Her mother developed bleeding during the early pregnancy. Her father used to imbibe enormous quantities of alcohol before conception. She has no fractures since her birth. The occipital frontal circumference was 35.0 cm (normal). Her extremities were obviously hypertrophic. She was diagnosed with right femoral tortuous *via* an ultrasonography examination before her birth. She has a normal older sister that shared the same mother but different father. The study obtained ethical approval from the Review Board of Tongji Hospital ethical committee, and a written informed consent was received.

#### Radiographic Examinations

The radiographic characteristics are quite classical and unique. Diaphyseal and metaphyseal modeling defects lead to tubular bone deformation and enlargement of the diaphyseal and metaphyseal part. She has facial and skeletal disfigurements, such as genu varus/valgus, bilateral cubitus valgus, and bilateral elbow extension limitation. Her skeletal legs have developed into tortuous shape like S-type but without length discrepancy. She was diagnosed with lateral dislocation of the patella for both legs. All of her long bones were tortuous and have developmental problems. The diaphysis of the long bones was wide and with thin cortexes, but the epiphysis is not as wide as the diaphysis, for example, neither femoral epiphysis is wide, and the growth plates were also very thin compared with her age. The second ossification center of the radial head did not appear.

##### Upper Limb

###### Humerus.

**Figure 2:**
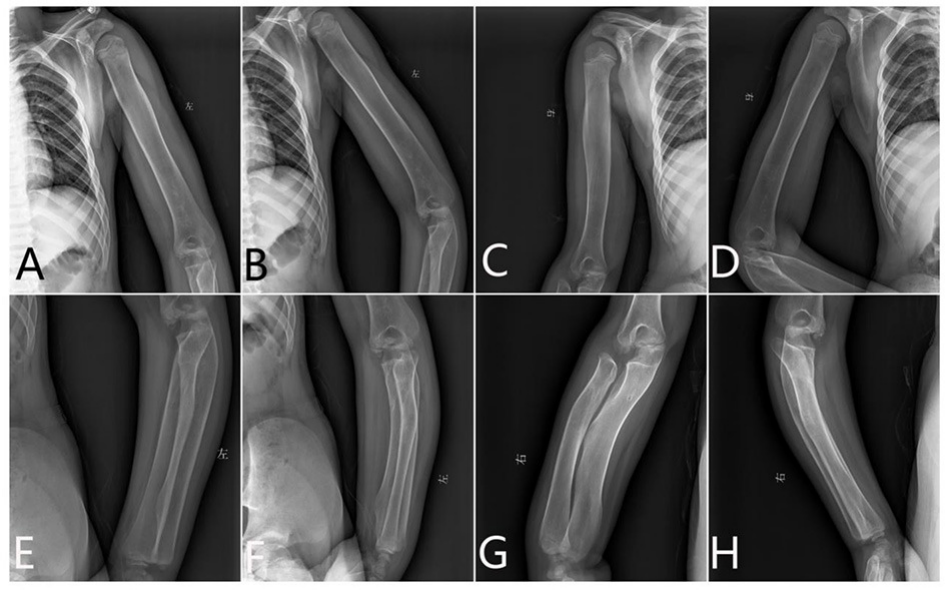
The x-ray radiography of the humerus, radius, and ulnas. **(A,B)** Left humerus, **(C,D)** right humerus, **(E,F)** left limb, and **(G,H)** right limb.

The metaphysis was not dilated. The diaphysis was wide and cylindrical and looks like a round rod shape. There was some thickened diaphyseal cortex, but was less than normal. Osteopenia was present. The distal end of the humerus lost its normal shape (Figure 2).

###### Radius and Ulnas.

The proximal end of the ulna was bulky and normally not modeled. The radius and ulnas were bowed. The radius head was pointed and beaklike, which did not match the lateral condyle of the humerus. It appears like the epiphysis is very small compared with the metaphysis (Figure 2).

###### Hand.

**Figure 3:**
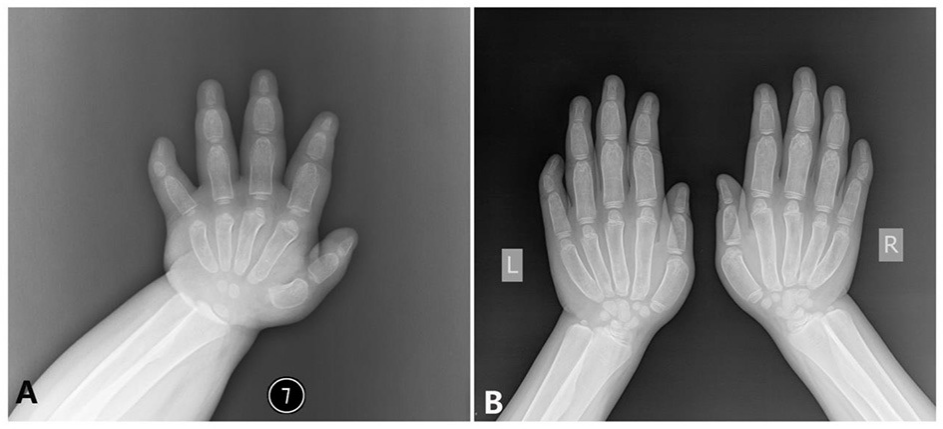
The x-ray radiography of the hands [shows short and wide phalanges and deformable bones; **(A)** age 1 year and 5 months, **(B)** age 6 years and 8 months].

The modeling abnormality of the tubular bones was greatest in the proximal and middle phalanges, which looks like a bullet shape. These bones were widest in the midportion of the diaphyses. A similar but less marked modeling abnormality was present in the metacarpals, but not in the distal phalanges. There is osteopenia and absence of the normal thick diaphyseal cortex, which makes all tubular bones of cortical thin. The epiphyses and carpal bones were smaller than the normal standards (Figure 3). However, the tip of each finger showed sharply pointed distal phalanges without changes after growth. The ossification centers of those carpal bones were quite small.

##### Lower Limb

###### Femur.

**Figure 4:**
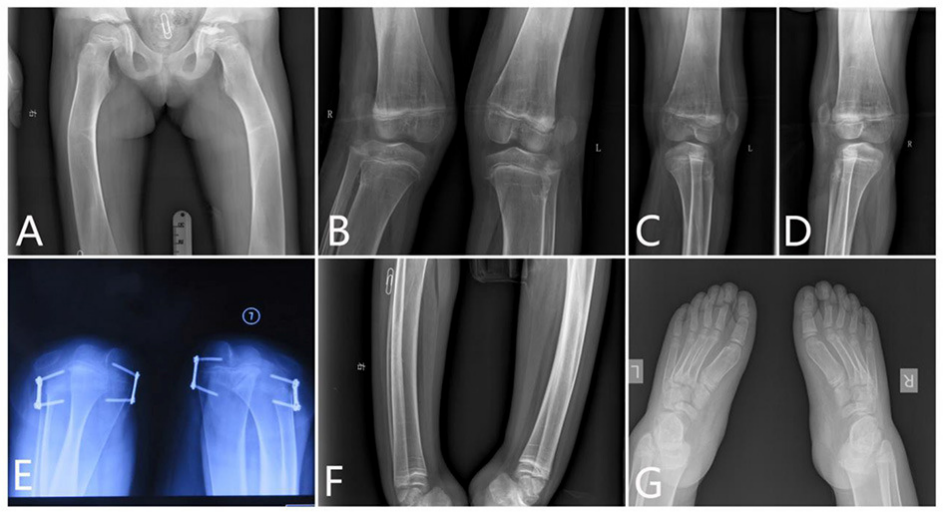
The x-ray radiography of the lower limbs. [**(A)** femurs, **(B–D)** knees, **(E)** shows lateral dislocation of the patella, **(F)** tibia, and **(G)** feet].

There was severe bilateral coxa valga. The femoral necks were wide. The cortex of the proximal diaphysis was thickened, but less than normal. Below this, the diaphysis did not have a normal shape but gradually expands to the metaphysis, which lacks a normal expansion area. The lower part of the femur had a thin cortex, and the entire bone was osteopenic and curved. The intercondylar fossa was shallow and flat (Figure 4).

###### Knee.

The x-ray of the knees showed lack of normal constriction of the metaphysis. The epiphysis developed quite small that did not match the size of the metaphysis. The patellae were located outside of the femur, which means a patella dislocation, and the patellae were quite small (Figure 4).

###### Patella.

The patellae were not located in the notch of the distal end of the femur. Lateral dislocation of the patella was apparent. The patella completely prolapsed outward (Figure 4).

###### Tibia and Fibula.

The tibia had a short mid-diaphyseal segment with almost normal cylindrical shape and cortical thickening. Above and below this segment, the bone became wider to the level of the growth plates. Metaphyseal flaring was absent. The cortical bone was thin in the proximal and distal thirds of the bone. The bone appeared osteopenic. Similar but less marked changes were present in the fibula (Figure 4). The shape of the proximal fibula is nearly normal. There were some Harris lines showing in the distal metaphyseal of the tibia. The growth plate of the distal tibia is not smooth.

###### Feet.

Her feet were rather big and flat. The ossification centers of those tarsals were rather small compared with the enlarged metatarsals. However, those phalanges of the toes were deformed and cylindrical shaped. The end digital phalanges were also deformed, which is different from her phalanges of the hands (Figure 4).

##### Trunk

###### Head.

From the x-rays of her head, we can see that her head is enlarged. The bone lamella of the parietal bone and occipital bone were rather thin, but the mandible was quite big and square shaped. Most important of all, there is no wormian bone located between the skull bones. A craniofacial CT scan revealed poor pneumatization of the mastoid process and enlargement of the mastoid process, but no multilocular tissue expansion developing in the maxilla and the mandible.

##### Chest

The clavicles were wide with the greatest expansion medially and laterally. The ribs were wide. The scapula was enlarged.

###### Abdomen and Pelvis.

**Figure 5:**
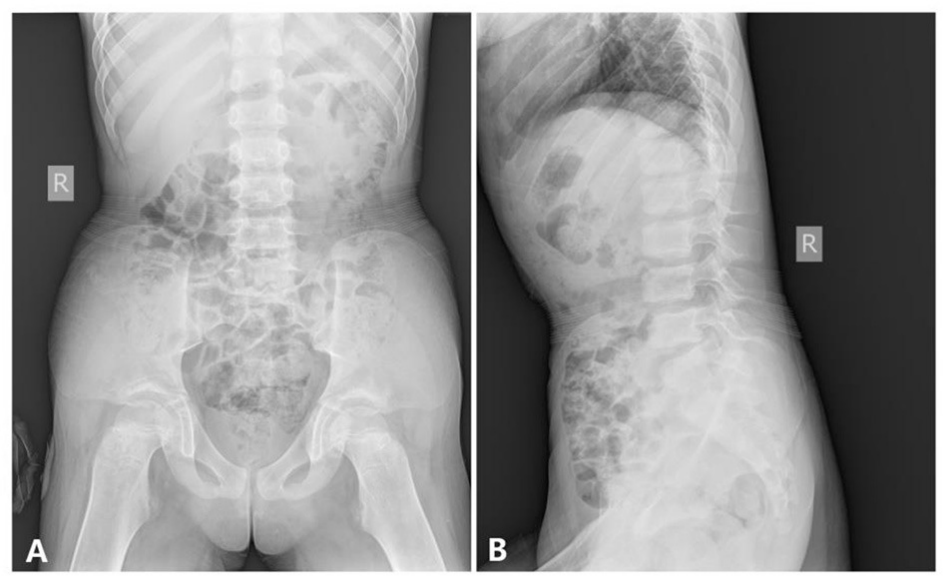
The x-ray radiography of the abdomen and the pelvis [age 6 years and 8 months, **(A)** anteroposterior film, **(B)** lateral film, shows].

The pelvic inlet was long and narrow in the frontal projection (the anteroposterior diameter of the pelvic inlet was increased), which looks like a pointed chin shape. The pelvic bones appeared immature without the normal modeling for age; thus, there was no supra-acetabular constriction in the ilia. The ilium was rather wide and looks like a cattail leaf fan (Figure 5).

###### Pituitary.

She received a pituitary MRI examination when she was 1.5 years old since her tall stature and megalakria acromegaly. The height of the pituitary was about 7 mm, which shows a little enlargement of the pituitary and a Rathke's fissure, but no pituitary adenomas were found.

###### Viscus.

Another CT examination showed no abnormalities in her lungs, heart, thyroid, liver, kidneys, pancreas, spleen, and adrenal glands.

### Examination at Birth

She was diagnosed with a special manifestation of osteopetrosis by x-ray after birth. She was diagnosed with wet lung disease of neonatal, macrosomia, high risk infant, osteopetrosis, and patent foramen ovale since birth. X-ray examination of the lower limbs showed the bone density of the lumbosacral vertebra, pelvis, bilateral femur, and tibiofibula to be increased, the femoral marrow cavity was unclear, the tibia and fibula bone cortex was thickened, the medullary cavity was narrowed, the bilateral femur and tibiofibula were curved, the lower limbs showed O-shaped leg changes, and the possibility of metabolic osteopathy was considered. Abdominal color Doppler ultrasound showed no abnormality. Patent foramen ovale was revealed by echocardiography.

### Laboratory Studies

Informed consent from the parents and approval from the local institutional review board were obtained for the studies. DNA was extracted from peripheral blood samples by standard procedures obtained from the patient and her parents. Mucopolysaccharidosis and Prader–Willi syndrome were excluded by means of Sanger sequencing. A whole-exome analysis was performed *via* use of the NovaSeq6000 platform (Illumina, San Diego, CA, USA) and SureSelectXT Human All Exon V6 (Agilent Technologies, Santa Clara, CA, USA), which provided 12 gigabases per sample. Approximately 84,000 variants were identified in the patient. The sequencing reads were aligned to the reference human genome sequence (hg19) using Burrows–Wheeler Aligner, and local realignment around the indels and base quality score recalibration were performed using the Genome Analysis Toolkit; duplicate reads were removed by Picard (http://picard.sourceforge.net). We assayed plasma levels of osteocalcin (OC), procollagen type 1 N-terminal propeptide (P1NP), and alkaline phosphatase as markers of bone formation, type I collagen cross-linked C-terminal telopeptide (CTX) as a marker of bone resorption, and parathormone (PTH).

### Surgery and Treatment

**Figure 6:**
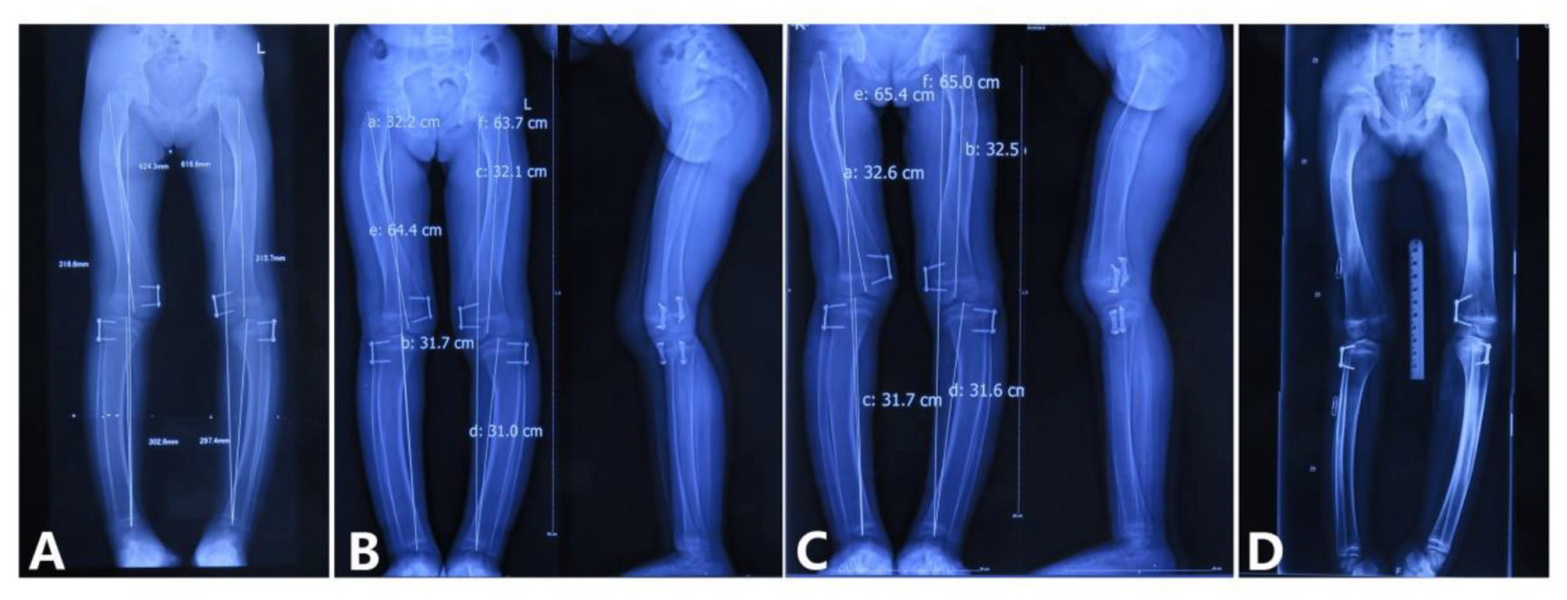
Correction of the lower limb deformity via the second harf epiphysiodesis surgery [shows lower limb deformity and tortuous lower limbs; **(A)** age 5 years and 3 months, 3 months after surgery; **(B)** age 5 years and 8 months, 9 months after surgery; **(C)** age 5 years and 11 months, 11 months after surgery; **(D)** age 6 years and 6 months, 1 year and 6 months after surgery].

Full-length radiographs of lower limbs showed a bending shape of her legs. She received twice harf epiphysiodesis surgeries in the Beijing Jishuitan Hospital to correct the leg deformity, in July 2016 and November 2018, respectively. Unfortunately, tortuous leg deformity is not corrected as intended, and this time, she came to our hospital to remove out the implants (Figure 6).

## Results

### Diagnosis

Craniotubular dysplasias (CTDs) are a heterogeneous group of genetic disorders of skeletal dysplasia, mainly involving the skull and the long bones, which is quite characteristic on the radiography and whose clinical and etiological classification is still much debated. The bone modeling problems usually lead to developmental genu varus/valgus and multiple fractures that are caused by bone fragility. The severe bone abnormalities result in technical problems, which hinder surgery in some extreme cases.

### Markers of Bone Turnover and Hormones

We assayed plasma levels of OC, P1NP, and alkaline phosphatase (ALP) as markers of bone formation, β-CrossLaps (β-CTX) as a marker of bone resorption, and PTH in the patient (Table 1). We also tested the plasma levels of calcium and phosphate for this patient (Table 1). The P1NP levels exceeded those of the age-related control values, which may be due to the increased synthesis and deposition rate of type I collagen (value 804.4 ng/ml, ref 8.53–64.32 ng/ml). OC levels were also higher than the reference values (value 184.6 ng/ml, ref 4.11–21.87 ng/ml). The level of ALP was elevated (value 387 U/L, ref <269 U/L). Together, the three osteoblast-derived metabolites seem to indicate a particularly active bone deposition process. The level of β-CTX was also higher than reference values (value 3.03 ng/ml, ref 0.068–0.68 ng/ml), which suggested an increasing bone resorption rate. The level of PTH was in the normal range (value 36.73 pg/ml, ref 15–65 pg/ml). The main results of the laboratory examination are listed in Table 1.

**Table 1:** The results of laboratory examination.

**Age**	**Birthday**	**1m**	**1y**	**1y1m**	**1y3m**	**1y5m**	**4y7m**	**5y10m**	**6y6m**
WBC (G/L)	18.88	8.30		7.17				5.14	5.42
N	77.30%	47.10%		56.4%				51.0%	44.4%
L	16.60%	36.10%		27.1%				38.1%	42.3%
Hb (g/L)	196	127		120.0				113	119.0
PLT (G/L)	325	440		251.0				281	263
Mon (G/L)	0.91	0.93		1.12				0.38	0.60
ALT (U/L)	23	14	25			22	14	14	11
AST (U/L)	26	36	42			34	29	25	20
ALP (U/L)	260	568	354				338	297	387
BUN (mmol/L)	3.78		2.30			2.08	2.96	3.1	3.82
Cr (umol/L)	76.6		39.5			25	45	41	47
UA (umol/L)	482.5		209.3			205.9	286	300	248
Ca (mmol/L)	2.23		2.42		2.50		2.34	2.3	2.34
P (mmol/L)	1.9		1.44		1.40		1.57	1.56	1.65
Mg (mmol/L)	0.78		0.95		0.92				0.8
GLU (mmol/L)			5.3			5.07	5.2	4.4	4.67
HbA1c					5.3%				
P1NP (ng/ml)					>1,200.00				804.4
CTX (ng/ml)					2,460.00		2.520	3.03
OC (ng/ml)							5.71		184.6
PTH (pg/ml)									36.73
**Age**	**1y5m**			**Age**			**4y7m**		
LH (mU/ml)	0.85			24hUFC (ug/24 h)			15.55		
FSH (mU/ml)	7.23			U-Ca (mmol/L)			1.29		
E2 (pg/ml)	13.79			U-P (mmol/L)			13.80		
ACTH (pg/ml)	102 (4PM)			24hU-Ca (mmol/24 h)			0.65		
COR (nmol/L)	142.0			24hU-P (mmol/24 h)			6.97		
**Age**	**6y6m**			**IGF-1(ng/ml)**			**51**		
GH (ng/ml)	0.55			1,25-OH VitD3 (pg/ml)			60.63		
IGF-1 (ng/ml)	60.5			25-OH VitD (ng/ml)			20.2		

*Red indicates upper than references. Green indicates lower than references*.

### Identification of the WRAP53 Deficiency

We used the BWA software (Burrows–Wheeler Aligner, version 0.7.12) to compare clean reads with the reference genome hg19 (http://hgdownload.cse.ucsc.edu/GOldenPath/hg19/bigZips/). The initial alignment results were in SAM format, and then the results were converted to BAM format and sorted by samtools software (Sequence Alignment/Map Tools, version 1.4). If the result of a sample contains multiple libraries, the fastq sequences of multiple libraries are merged first, and then the repeated sequences are marked and removed by Picard (picard-tools, version 1.57). Finally, the basic data information statistics and map comparison statistics are carried out. The results are listed as follows (Table 2).

**Table 2:** Quality control results compared with reference human genome.

**Sample from**	**Patient**	**Father**	**Mother**
Clean bases	11,038,684,484	11,622,109,710	11,627,779,498
Clean reads	80,720,060	84,154,772	84,515,422
Mapped reads	76,360,007 (94.60%)	79,579,851 (94.56%)	80,031,255 (94.69%)
Duplicate reads	13,913,098 (18.22%)	13,515,722 (16.98%)	13,918,750 (17.39%)
Unique reads	62,446,909 (81.78%)	66,064,129 (83.02%)	66,112,505 (82.61%)
Reads uniquely mapped to target	43,452,503	43,920,517	46,520,499
Reads uniquely mapped to genome	62,446,909	66,064,129	66,112,505
Total bases on target	60,456,963	60,456,963	60,456,963
Total bases near target	75,840,481	75,840,481	75,840,481
Total sequences on target (Mb)	4,977.45	5,069.27	5,351.66
Total sequences near target (Mb)	1,216.6	1,284.52	1,313.09
Total effective yield (Mb)	8,477.75	9,055.76	9,030.68
Fraction of effective bases on target	58.71%	55.98%	59.26%
Fraction of effective bases on or near target	73.06%	70.16%	73.80%
Fraction of uniquely mapped on target	69.58%	66.48%	70.37%
Average sequencing depth on target	82.33	83.85	88.52
Average sequencing depth near target	16.04	16.94	17.31
Average insert size of the library	189.73	193.72	191.63
Base covered on target	59,182,242	59,263,924	59,199,883
Coverage of target region	97.89%	98.03%	97.92%
Base covered near target	71,077,519	71,678,056	71,406,996
Coverage of near target	93.70%	94.50%	94.20%
Mismatch rate in target	0.35%	0.35%	0.30%
Mismatch rate in total	0.35%	0.36%	0.31%
Fraction of target covered at least 4 ×	97.52%	97.65%	97.57%
Fraction of target covered at least 10 ×	96.76%	96.92%	96.91%
Fraction of target covered at least 20 ×	93.70%	93.89%	94.37%
Fraction of target covered at least 50 ×	66.65%	67.38%	70.40%

**Figure 7:**
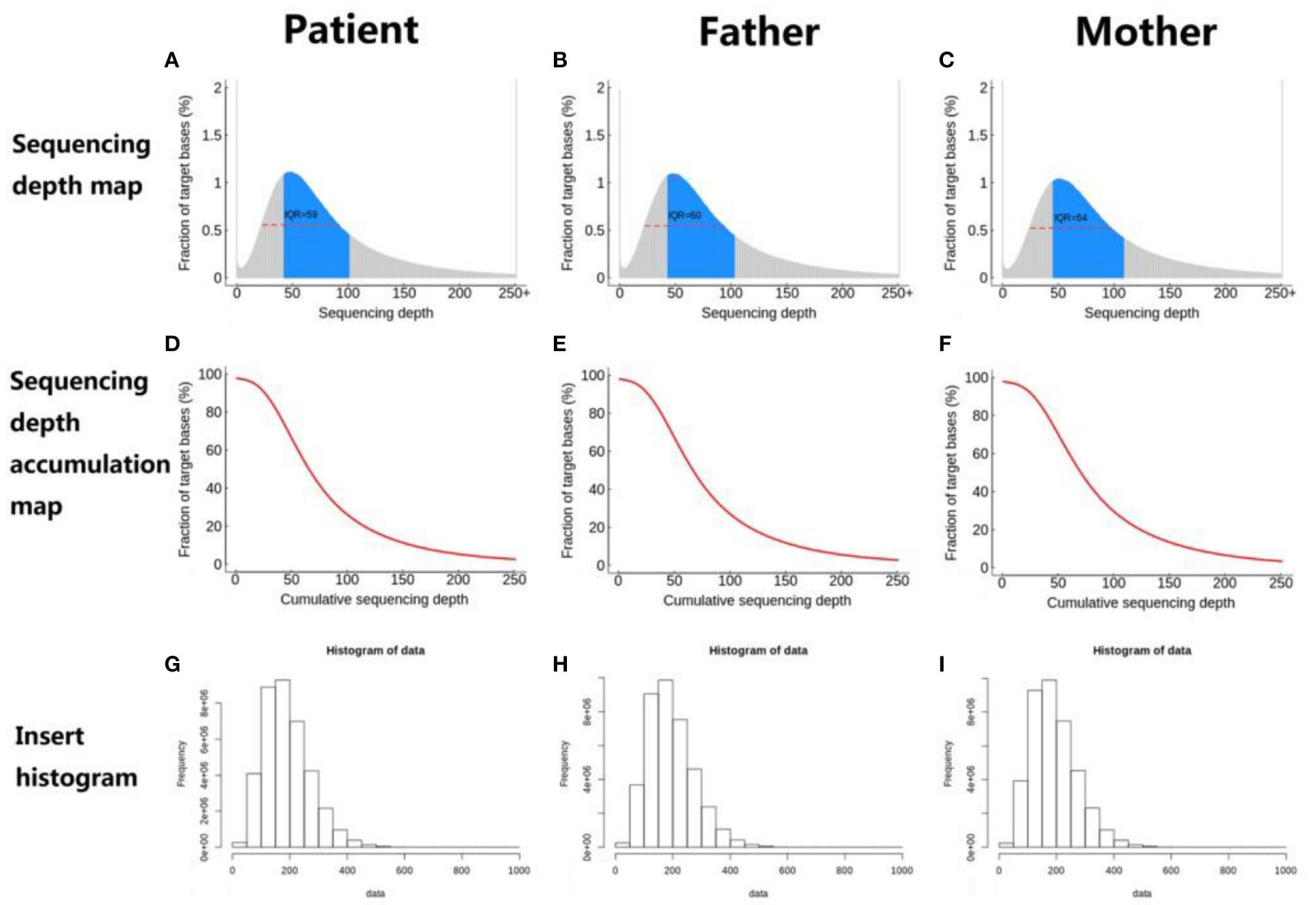
**(A–I)** Sequencing coverage distribution.

The distribution of sequencing coverage is an important indicator to measure the homogeneity of sequencing. Therefore, we counted the distribution of sequencing coverage and visually displayed it, as shown in the following figures (Figure 7).

**Figure 8:**
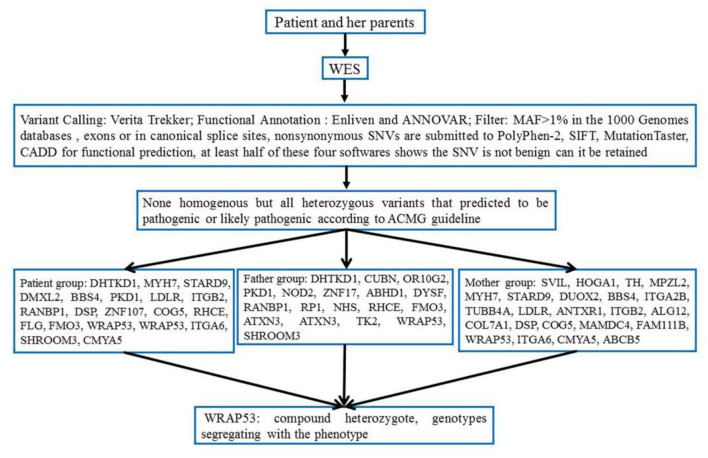
Flowchart of this whole-exome sequencing (WES) study and gene identification.

Based on the comparison results, the Verita Trekker mutation system (BerryGenomics) was used to detect SNV and indel mutation sites, and xhmm was used to detect CNV. According to the enliven annotation system (reference to ClinVar, OMIM, NCBI, ClinGene, 1000 genome, and so on), SNV, indel, and CNV are annotated to determine the functional region, gene information, synonymous/non-synonymous mutation, and prediction of amino acid toxicity of variant sites. We revealed several copy number variations (CNV) but all identified as VUS or benign-type according to the ACMG guideline (Figure 8).

SNV is annotated from five aspects, including basic information of the mutation site, gene and region information, normal person database (frequency), conservative (harmful) prediction annotation, and gene function and pathway annotation, which are according to the ACMG guideline (Richards et al., 2015). Approximately 84,000 variants were identified in the patient. Filtering of the exome-sequencing data from the patient and her parents resulted in the retention of none homogenous, but all heterozygous, variants that were predicted to be pathogenic or likely pathogenic according to the ACMG guideline. Patient group: DHTKD1, MYH7, STARD9, DMXL2, BBS4, PKD1, LDLR, ITGB2, RANBP1, DSP, ZNF107, COG5, RHCE, FLG, FMO3, **WRAP53, WRAP53**, ITGA6, SHROOM3, and CMYA5. Father group: DHTKD1, CUBN, OR10G2, PKD1, NOD2, ZNF17, ABHD1, DYSF, RANBP1, RP1, NHS, RHCE, FMO3, ATXN3, ATXN3, TK2, **WRAP53**, and SHROOM3. Mother group: SVIL, HOGA1, TH, MPZL2, MYH7, STARD9, DUOX2, BBS4, ITGA2B, TUBB4A, LDLR, ANTXR1, ITGB2, ALG12, COL7A1, DSP, COG5, MAMDC4, FAM111B, **WRAP53**, ITGA6, CMYA5, and ABCB5 (Table 3).

**Table 3:** Family sequencing for genotype of the WRAP53 gene.

**Level**	**Evidence**	**Ref**	**Alt**	**ExonicFunc**	**dbsnp**	**Exon**	**hgvs.c**	**hgvs.p**	**Patient**	**Father**	**Mother**
Benign	ba1 bp6 bp4	C	G	Non-synonymous SNV	rs2287499	Exon1	c.202C>G	p.R68G	Het	Het	Hom
Benign	ba1 bp6 bp7	C	T	Synonymous SNV	rs2287498	Exon2	c.450C>T	p.F150F	Het	Het	Hom
Benign	pm1 ba1 bp6 bp4	C	G	Non-synonymous SNV	rs7640	Exon10	c.1565C>G	p.A522G	Het	Het	Hom
Likely pathogenic	pvs1 pm2	TCTC	–	Frameshift deletion	–	Exon1	c.224_227del	p.L75Pfs*14	Het	–	Het
Likely pathogenic	pvs1 pm2	G	–	Frameshift deletion	rs1085307105	Exon10	c.1564del	p.A522Rfs*26	Het	Het	–
**dbsnp**	**1,000g2015** **aug_all**	**1,000g2015** **aug_eas**	**BerryAF**	**ExAC_ALL**	**ExAC_EAS**	**gnomAD_exome** **_ALL**	**gnomAD_exome_** **AFpopmax**	**gnomAD_** **exome_EAS**
rs2287499	0.406749	0.2996	0.266271	0.212	0.294595	0.199237	0.773168	0.309216
rs2287498	0.222244	0.2996	0.213251	0.133	0.293566	0.130306	0.308395	0.308395
rs7640	0.511182	0.4077	0.187613	0.313	0.412544	0.299461	0.824941	0.420597
							0.000163114	.
rs1085307105				0.0001483			0.00119956	.

**Figure 9:**
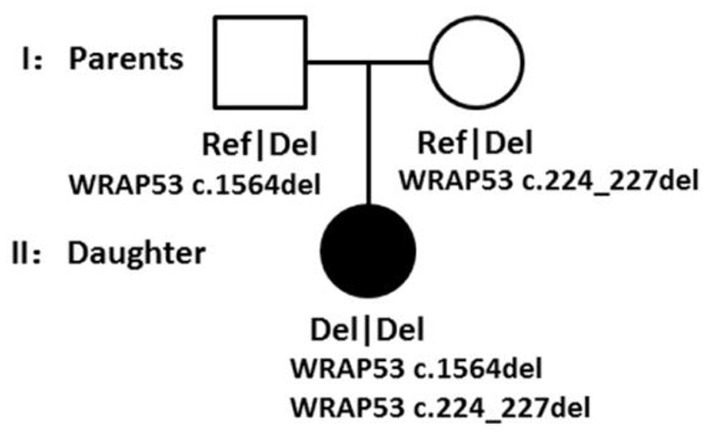
Family pedigree showing the genotypes segregating with the phenotype.

Though all of those mutations were heterozygous, there is a compound heterozygote that is WRAP53, and more importantly, one mutation was received from her father and another mutation from her mother. Each mutation is a type of frameshift deletion, which affected the translation and the protein function. Since the patient got each mutated gene from her parents, this will lead to her inability to translate normal mRNA of the WRAP53 gene (Figure 9).

Copy number variations (CNVs) are structural variations of a genome, which can be divided into two levels: microscopic level and submicroscopic level. Microscopical genomic structural variation mainly refers to the chromosomal aberrations visible under the microscope, including aneuploid, deletion, insertion, inversion, translocation, fragile site, and other structural variations. Sub microlevel genomic structural variation refers to the genomic structural variation with the length of the DNA fragment ranging from 1 kb to 3 mb, including deletion, insertion, duplication, rearrangement, inversion, DNA copy number change, etc., which are collectively referred to as CNV. Here, we also tested the CNVs (Table 4). However, we find no CNV associated with wrap53 on chromosome 17.

**Table 4:** Copy number variations detection results.

**Level**	**CNV**	**Type**	**Chr**	**Size**	**CopyRatio**	**Cytoband**	**Aneuploidy**	**Gene**
Polymorphism	chr1:668342–672153_dup	Duplication	chr1	3.81Kb	2.72	p36.33	–	
Polymorphism	chr1:879059–899623_dup	Duplication	chr1	20.57Kb	1.16	p36.33	–	SAMD11, NOC2L, KLHL17
Polymorphism	chr1:1221280–1249356_dup	Duplication	chr1	28.08Kb	1.14	p36.33	–	SCNN1D, ACAP3, PUSL1, INTS11
Polymorphism	chr1:1396089–1451457_dup	Duplication	chr1	55.37Kb	1.12	p36.33	–	ATAD3C, ATAD3B, ATAD3A
Polymorphism	chr6:29910345–29913411_dup	Duplication	chr6	3.07Kb	1.25	p22.1	–	HLA-A
VUS	chr9:139396643–139581860_dup	Duplication	chr9	0.19Mb	1.16	q34.3	–	NOTCH1, EGFL7, AGPAT2
Polymorphism	chr11:1031548–1090952_dup	Duplication	chr11	59.41Kb	1.17	p15.5	–	MUC6, LOC101927503, MUC2
Polymorphism	chr12:132391306–132401133_dup	Duplication	chr12	9.83Kb	1.12	q24.33	–	ULK1
Polymorphism	chr14:105408841–105419660_dup	Duplication	chr14	10.82Kb	1.1	q32.33	–	AHNAK2
Polymorphism	chr14:106054389–106208551_dup	Duplication	chr14	0.15Mb	1.39	q32.33	–	
VUS	chr19:4510405–4513806_dup	Duplication	chr19	3.40Kb	1.14	p13.3	–	PLIN4
Polymorphism	chr22:18020069–18102275_dup	Duplication	chr22	82.21Kb	1.52	q11.21	–	CECR2, LOC101929372, SLC25A18, ATP6V1E1
Polymorphism	chr22:25713983–25961264_dup	Duplication	chr22	0.25Mb	1.42	q11.23-q12.1	–	LRP5L, GRK3
VUS	chr1:152595355–152785061_del	Deletion	chr1	0.19Mb	0.88	q21.3	–	LCE3A, LCE2D, LCE2C, LCE2B, LCE2A, LCE4A, C1orf68, KPRP, LCE1F, LCE1E, LCE1D, LCE1C, LCE1B
Polymorphism	chr5:180376224–180429913_del	Deletion	chr5	53.69Kb	0	q35.3	–	BTNL8, BTNL3
Polymorphism	chr10:124343759–124351967_del	Deletion	chr10	8.21Kb	0.51	q26.13	–	DMBT1
Polymorphism	chr11:55370904–55406945_del	Deletion	chr11	36.04Kb	0	q11	–	OR4C11, OR4P4
Polymorphism	chr12:9455172–9464465_del	Deletion	chr12	9.29Kb	0.82	p13.31	–	
Polymorphism	chr12:52840964–52909065_del	Deletion	chr12	68.10Kb	0.92	q13.13	–	KRT6B, KRT6C, KRT6A, KRT5

Since the parental consanguinity in the families with craniometadiaphyseal dysplasia reported by Langer et al. (1991) and Santolaya et al. (1998) is consistent with autosomal recessive inheritance, 24,061 variants were selected for their gene functions as exonic and splicing. We get 12,298 variants after removing the synonymous SNV variants. Thus, we selected all of the homozygous mutations from those variants detected and resulted in 4,961 items. We also selected the homozygous mutations in those exonic and splicing, and excluded out those synonymous SNV variants for her biological father and mother. Because her father and mother all have the normal phenotype. We made a kind of elimination of those same homozygous mutations with her parents. Eliminated by her father's same homozygous mutations, we get 793 variants. Eliminated by her mother's same homozygous mutations, we get 902 variants. Combining them together, we get 331 variants (260 genes). Then we made a Scientific Literature Retrieval through the Pubmed Database. We used the “variant gene name” plus “bone” as the search strategy. All of the genes were divided by the retrieved results into three groups: group highly correlated, group correlated, and group uncorrelated. The relationship mainly depends on the effect of those genes on osteoblasts and osteoclasts. Filtering of the exome-sequencing data from patients resulted in the retention of 19 homozygous variants that are in the group that are highly correlated: ACKR2, ACTN3, ADARB2, FNDC3B, HDAC5, IL15RA, MMP9, PARG, PDZD7, SELP, SH3BP2, SLC36A3, SPRY2, TDGF1, TLL1, TLN1, TSPEAR;KRTAP10-5, VWC2, and ZNRF3. Only PARG, SH3BP2, and ZNRF3 genes were identified as the VUS group, and all of what were left were divided into the benign group. Thus, we need to further analyze PARG, SH3BP2, and ZNRF3. Since ZNRF3 is a WNT inhibitor, it may be another gene most likely related to the bone development and metabolism—diaphyseal and metaphyseal modeling defects.

## Discussion

Craniotubular dysplasias (CTDs) are heterogeneous group of genetic bone disorders characterized by modeling errors of the craniofacial and tubular bone structure, whose clinical and etiological classification is still much debated (Gorlin, 1994). The radiological hallmarks include craniofacial hyperostosis and metadiaphyseal under modeling of the tubular bones (Nishimura et al., 1997). CTD contains a large group of bone diseases that include craniometaphyseal dysplasia (OMIM 123000, 218400), craniodiaphyseal dysplasia (OMIM 122860), frontometaphyseal dysplasia (OMIM 305620, 617137), craniometadiaphyseal dysplasia (OMIM 269300), osteopetrosis, dysosteosclerosis (OMIM 224300), oculodentoosseous dysplasia (OMIM 164200, 257850), osteopathia striata (OMIM 300373), autosomal dominant otosclerosis (endosteal hyperostosis, Worth type), Van Buchem disease (OMIM 239100), sclerosteosis (OMIM 269500, 614305), diaphyseal dysplasia Camurati–Engelmann (OMIM 131300), hyperphosphatasemia, and Stanescu osteosclerosis syndrome. The application of massively parallel sequencing technology in the field of skeletal disorders has boosted the discovery of the underlying genetic defect in many such diseases. It has also resulted in the delineation of new clinical entities and the identification of genes and pathways that had not previously been associated with skeletal disorders (Yang et al., 2019). Filtering of the exome-sequencing data from patients diagnosed as Pyle's disease resulted in the discovering of the underlying genetic defect as SFRP4 deficiency (Kiper et al., 2016). However, only four cases of craniometadiaphyseal dysplasia have been reported to date, and none of the studies were done to determine the candidate genes or loci or even perform genome-wide analysis; thus, the molecular etiology is still unknown.

### Clinical Diagnosis and Differential Diagnosis From Pyle's Disease

Craniometadiaphyseal dysplasia (CRMDD) is characterized clinically by macrocephaly with frontal prominence, prominent mandible, dental hypoplasia, and increased bone fragility. Schwarz (1960) presented the first case of craniometadiaphyseal dysplasia as an example of craniometaphyseal dysplasia with short stature, frontal bossing, prominent mandible, and dental caries in an 18-year-old girl (patient 1). Langer et al. (1991) described the same on a brother and sister later (patient 2 and 3). Langer et al. (1991) called this condition craniometadiaphyseal dysplasia (CRMDD), wormian bone type, and distinguished it from craniometaphyseal dysplasia (CMDR, OMIM 218400), and Langer et al. (1991) first described the radiographic characteristics of the craniometadiaphyseal dysplasia as wide long tubular bones without normal metaphyseal flaring and wide short tubular bones without normal diaphyseal constriction. Santolaya et al. (1998) described a 4-year-old Moroccan boy (patient 4), the first child of first-cousin parents, with craniometadiaphyseal dysplasia, wormian bone type. The patient had a large head with a prominent forehead and skull changes showing multiple wormian bones. Dhar et al. (2010) provided a 25-year follow-up of the brother reported at the age of 8 years by Langer et al. (1991) and reviewed other reported cases. We report here on a 6-year follow-up of craniometadiaphyseal dysplasia in a 6-year-old Chinese girl. Mucopolysaccharidosis and Prader–Willi syndrome were excluded by means of Sanger sequencing when she was 1 year and 6 months. A whole-exome analysis also confirmed this. IGF-1 and GH hormone test, MRI examination of the pituitary, and preformation of CT on the abdomen has also ruled out growth developmental disorders caused by the endocrine system. Serum calcium and phosphate level were normal. Serum PTH level was also in normal line. The P1NP levels exceeded reference values, possibly resulting from an increased rate of type I collagen synthesis and deposition. Osteocalcin levels were also higher than reference values, which seem to indicate a particularly active bone deposition process. Taken together, those bone markers tend to indicate a high bone turnover condition. The levels of bone-specific alkaline phosphatase were normal.

She had no history of bone fractures, but she has bowing of the lower limbs, bilateral cubitus valgus with dislocated radial heads, coxa valga deformity, and deformities of the pelvis and elbows. She presented with a large head, prominent forehead and underjaw, high-arched palate, and hypoplastic teeth; wide long tubular bones without normal metaphyseal flaring; wide short tubular bones without normal diaphyseal constriction; and with actual diaphyseal expansion; and wide ribs and clavicles. We can easily differentiate craniometadiaphyseal dysplasia from Pyle's disease by x-ray examination of the tubular bones. Pyle's disease is characterized by unusual facies and club-shaped metaphyseal flaring of the long bones. On radiographs of patients with craniometadiaphyseal dysplasia, the wide long tubular bones were without normal metaphyseal flaring, the wide short tubular bones were without normal diaphyseal constriction, and the tubular bones were with diaphyseal expansion, which were caused by diaphyseal and metaphyseal modeling defects, but the underlying genetic defect has proved elusive.

### Genetic Diagnosis and Inheritance Analysis

According to the WES result, all of those pathogens of likely pathogenic mutations were heterozygous, but there is a compound heterozygote that is gene WRAP53, and more importantly, one mutation was received from her father and another mutation from her mother. Here, we also tested the CNVs in the case of gene translocation. However, we do not find any CNV of the chromosome 17 on which the gene WRAP53 is located. Therefore, we show for the first time that biallelic frameshift deletion mutations in WRAP53 result in craniometadiaphyseal dysplasia. The predicted structure of the WRAP53β protein includes an unstructured N-terminal region (including a proline-rich section), a domain with seven WD40 repeats, followed by a short C-terminal extension containing a glycine-rich region (Mahmoudi et al., 2009). The identified mutation was a c.224_227del in exon 1, with a premature stop codon in exon 1 leading to a predicted 89 amino acid sequence, and another identified mutation was a c.1564del in exon 10, with a premature stop codon in exon 10 leading to a predicted 548 amino acid sequence with amino acid sequence change.

Wrap53 is a natural p53 antisense transcript, which regulates the level of endogenous p53 mRNA and induces p53 protein by targeting the 5′ untranslated region of p53 mRNA (Mahmoudi et al., 2009). Knockdown of Wrap53 significantly reduces p53 mRNA and protein expression, and this is not due to the block of transcription but instead occurs at the posttranscriptional level (Farnebo, 2009). The WRAP53 gene gives rise to a p53 antisense transcript that regulates p53. This gene also encodes a protein that directs small Cajal body-specific RNAs to Cajal bodies. Cajal bodies are nuclear organelles involved in diverse functions such as processing ribonucleoproteins, which are very important for splicing (Mahmoudi et al., 2010). WRAP53β plays essential roles in localizing various factors to the nuclear organelles known as Cajal bodies, to telomeres, and to DNA double-strand breaks and is important in connection with the regulation of nuclear architecture, telomere elongation, and DNA repair (Henriksson et al., 2014; Bergstrand et al., 2020).

In two unrelated patients with dyskeratosis congenital (DC), Zhong et al. (2011) identified compound heterozygous mutations in the WRAP53 gene (612,661.0001–612,661.0004). Each unaffected parent was heterozygous for one of the mutations. To date, ACD, CTC1, DKC1, NHP2, NOP10, PARN, RTEL1, TERC, TERT, TINF2, and WRAP53 are the genes in which pathogenic variants are known to cause dyskeratosis congenita (DC) and result in very short telomeres (Savage and Alter, 2009). The hereditary disorder dyskeratosis congenita, and its most severe form the Hoyeraal–Hreidarsson syndrome (HHS), are associated with severely shortened telomeres and a variety of clinical symptoms, including fibrosis in the lung and liver, bone marrow failure, developmental defects, and cancer (mainly hematological and head and neck malignancies), as well as a classical triad of mucocutaneous features (abnormal skin pigmentation, oral leukoplakia, and nail dystrophy) (Savage and Alter, 2009; Kelmenson and Hanley, 2017). Bergstrand et al. (2020) showed for the first time that biallelic mutations in WRAP53 result in HHS. WRAP53β plays essential roles in localizing various factors to the nuclear organelles known as Cajal bodies, to telomeres, and to DNA double-strand breaks and is thus important in connection with the regulation of nuclear architecture, telomere elongation, and DNA repair. The classic triad may not be present in all individuals. The disease is heterogeneous at the genetic and clinical levels. Here, we reported a case of a patient also with compound heterozygous mutations in the WRAP53 gene, and this patient presented with one of the classic DC symptoms—nail dystrophy, which is one of the diagnostic cutaneous triad for DC. She also has teeth problems and osteoporosis, which is listed in additional features of DC (Savage and Alter, 2009). Those clinical findings match the disease that was caused by the disruption of telomerase trafficking with WRAP53 mutation to some extent. However, further studies are needed to explore the cellular mechanisms of the abnormal bone architecture in WRAP53-null mice.

## Conclusions

Here, we reported the radiographic changes of the patient at her birthdate that proved this rare disease to be a kind of congenital problem, and by reviewing her clinical case records, this disease was proved to be related with bone modeling—radiographic changes looks like osteopetrosis, but later, the x-ray examination showed absence of normal metaphyseal flaring and diaphyseal constriction, showed diaphyseal widening of the long tubular bones with wide undermineralized metaphyses, widened ribs and clavicles and broadening of short tubular bones with increased transparency and thin cortices, and showed quite a small secondary ossification center that developed from cartilage. While it is useful to make a diagnosis for those suspected cases by a hand radiography examination for typical bullet-shaped phalanges. There are several marked radiographic characteristics: (1) bullet-shaped phalanges, (2) pointed chin-shaped, long and narrow pelvic inlet, absence of supra-acetabular constriction, (3) round rod-shaped long tubular bones, (4) prominent aiploic mastoid, (5) bending-shaped limb, genua varus and genu varum, and (6) congenital dislocation of the elbow. Here, we did not find any wormian bone in her head by CT and x-ray examination. Thus, we would like to present our case as a new subtype of craniometadiaphyseal dysplasia, that is, without wormian bone-type, and in our case, there are several typical clinical characteristics: (1) macrocephaly and wide jaw, (2) avatar elf-shaped ears and pointed and protruding ears, (3) hypertrophy of the limbs, (4) flat feet and giant hand phenomenon, (5) nail dystrophy, (6) limb deformity, (7) high arched palate, (8) superficial hemangiomas, and (9) tall stature and intellectual disability.

The treatment of limb bending deformity by harf epiphysiodesis surgery tends to fail at last. It may be caused by diaphyseal and metaphyseal modeling defects, which lead to insensitivity to the stiffness mechanical stimulation. Of course, it can be a result of the failing of the controlling bone metabolism answered to the stiffness mechanical stimulation.

## Data Availability Statement

The original contributions presented in the study are included in the article, further inquiries can be directed to the corresponding author.

## Ethics Statement

The studies involving human participants were reviewed and approved by the Review Board of Tongji Hospital ethical committee. Written informed consent to participate in this study was provided by the participants' legal guardian/next of kin. Written informed consent was obtained from the minor(s)' legal guardian/next of kin for the publication of any potentially identifiable images or data included in this article.

## Author Contributions

J-PH, YH, and X-LW performed the research and analyzed the data. JX, C-LJ, X-YM, J-CG, J-FS, and J-XF analyzed the data. J-PH and X-LW designed the study. YH, J-PH, and X-LW supervised the study. YH and J-PH wrote the paper. All authors read and approved the final manuscript.

## Conflict of Interest

The authors declare that the research was conducted in the absence of any commercial or financial relationships that could be construed as a potential conflict of interest.

## Publisher's Note

All claims expressed in this article are solely those of the authors and do not necessarily represent those of their affiliated organizations, or those of the publisher, the editors and the reviewers. Any product that may be evaluated in this article, or claim that may be made by its manufacturer, is not guaranteed or endorsed by the publisher.
